# Theoretical Explorations Generate New Hypotheses About the Role of the Cartilage Endplate in Early Intervertebral Disk Degeneration

**DOI:** 10.3389/fphys.2018.01210

**Published:** 2018-09-19

**Authors:** Carlos Ruiz Wills, Baptiste Foata, Miguel Á. González Ballester, Jaro Karppinen, Jérôme Noailly

**Affiliations:** ^1^BCN MedTech, Department of Information and Communication Technologies, Universitat Pompeu Fabra (UPF), Barcelona, Spain; ^2^Institute for Bioengineering of Catalonia (IBEC), Barcelona, Spain; ^3^Catalan Institution for Research and Advanced Studies (ICREA), Barcelona, Spain; ^4^Medical Research Center Oulu, Oulu University Hospital and University of Oulu, Oulu, Finland; ^5^Center for Life Course Health Research, Oulu University Hospital, Oulu, Finland; ^6^Finnish Institute of Occupational Health, Oulu, Finland

**Keywords:** intervertebral disk degeneration, cartilage endplates, composition-dependent tissue permeability, finite elements simulations, disc cell nutrition, indirect mechanotransduction

## Abstract

Altered cell nutrition in the intervertebral disk (IVD) is considered a main cause for disk degeneration (DD). The cartilage endplate (CEP) provides a major path for the diffusion of nutrients from the peripheral vasculature to the IVD nucleus pulposus (NP). In DD, sclerosis of the adjacent bony endplate is suggested to be responsible for decreased diffusion and disk cell nutrition. Yet, experimental evidence does not support this hypothesis. Hence, we evaluated how moderate CEP composition changes related to tissue degeneration can affect disk nutrition and cell viability. A novel composition-based permeability formulation was developed for the CEP, calibrated, validated, and used in a mechano-transport finite element IVD model. Fixed solute concentrations were applied at the outer surface of the annulus and the CEP, and three cycles of daily mechanical load were simulated. The CEP model indicated that CEP permeability increases with the degeneration/aging of the tissue, in accordance with recent measurements reported in the literature. Additionally, our results showed that CEP degeneration might be responsible for mechanical load-induced NP dehydration, which locally affects oxygen and lactate levels, and reduced glucose concentration by 16% in the NP-annulus transition zone. Remarkably, CEP degeneration was a condition sine-qua-non to provoke cell starvation and death, while simulating the effect of extracellular matrix depletion in DD. This theoretical study cast doubts about the paradigm that CEP calcification is needed to provoke cell starvation, and suggests an alternative path for DD whereby the early degradation of the CEP plays a key role.

## Introduction

One of the principal causes of intervertebral disk (IVD) degeneration (DD) in the lumbar spine is suggested to be the alteration of the nutrient supply to disk cells (Urban and Roberts, 2003), which leads to local low levels of oxygen and glucose, and high levels of lactate, i.e., acidic pH. Whereas low oxygen tension and pH might affect the synthesis and maintenance of the disk extracellular matrix (ECM) (Urban and Roberts, 2003), they may also trigger the expression of enzymes, e.g., matrix metalloproteinases that would actively degrade this matrix (Neidlinger-Wilke et al., 2012). Altogether, these effects could accelerate DD. Cell nutritional stress affects ECM turnover in interaction with the mechanical loads transmitted through the tissues, via mechano-transport mechanisms. This phenomenon has been referred to as indirect mechanotransduction (Iatridis et al., 2006), and evidences at the cell level strongly suggest that the latter is actually more influent than direct mechanotransduction (Neidlinger-Wilke et al., 2012).

Nutrients can reach IVD cells from the peripheral blood supply, either through the outer annulus fibrosus (AF) or through the cartilage endplate (CEP) (Urban et al., 2004; Shankar et al., 2009), adjacent to the vertebral subchondral bone, i.e., the bony endplate (BEP). The solutes such as oxygen and glucose are transported into the disk through the CEP, and their availability is regulated by the bone marrow contact channels that cross the BEP (Benneker et al., 2005a,b). Early experimental results obtained by Nachemson et al. (1970) suggested that CEP calcification interferes with the nutrient supply. Nevertheless, Bernick and Cailliet (1982) reported that CEP calcification would occur progressively over several decades from about 40 years of age on, and theoretical models insinuate that severe nutrient deprivation within the disk requires advanced stages of contact channel occlusion, i.e., 40–50% of occlusion (Malandrino et al., 2014b). According to Benneker et al. (2005a), such degree of occlusion would occur with advanced to severe DD. Hence, CEP calcification is unlikely to be involved in early DD processes.

Yet, during disk aging or degeneration, the CEP clearly undergoes composition changes (Fields et al., 2015) that are likely to affect the solute transport properties of the tissue (Roberts et al., 1996; DeLucca et al., 2016). Furthermore, the CEP possesses sharp gradients of composition from the nucleus pulposus (NP) to the BEP (Roberts et al., 1989), in terms of water, proteoglycans, and collagen contents, and the main function of such gradients remains unclear. In general, the specific influence of CEP composition on the transport of nutrients into the IVD remains underexplored to our knowledge. While calcification would refer to sclerosis, the changes in water and macromolecule contents would refer to earlier degenerative changes, explored in the current study.

Direct observations of degeneration-dependent mechanisms of nutrient transport into the IVD and of the consequences thereof on disk cell nutrition are difficult to achieve *in vivo* and *in vitro*. Hence, numerical models are regarded as a promising tool to complement the experimental results (Malandrino et al., 2015a). Inspired by the seminal works of Nachemson et al. (1970) and Benneker et al. (2005a) about endplate solute conductance and bone marrow contact channel occlusion, CEP calcification was simulated *in silico* by reducing by 40–50% the tissue water content or diffusivity. The theoretical studies of Mokhbi Soukane et al. (2009) and Jackson et al. (2011) showed that simulating CEP calcification led to a reduction in glucose concentration in human lumbar IVD models, and the NP was the most affected disk region. Shirazi-Adl et al. (2010), Galbusera et al. (2011), Zhu et al. (2012), and Malandrino et al. (2014b) further predicted disk cell viability reductions as a possible consequence of CEP calcification. Under the effect of external mechanical loads, cell viability was mostly affected in the peripheral NP, nearby the AF-NP transition zone (TZ). The numerical study of Malandrino et al. (2014a) additionally suggested that BEP morphological changes with degeneration had a negligible effect on solute transport through the full endplate, but highlighted the possible importance of the CEP in DD.

Nevertheless, there is no consensus about the way to simulate CEP degenerative changes and the effect thereof on the rest of the IVD. In terms of fluid transport, Riches and McNally (2005) used a theoretical model and studied the influence of CEP permeability reduction on disk mechanical behavior. They found that CEP permeability would locally affect fluid movements and improve convective nutrition when CEP permeability is lower than disk tissue permeability. Ayotte et al. (2000, 2001) suggested that the gradient of mechanical properties between the CEP and the bone generate a direction-dependent effective permeability of the CEP. Furthermore, preliminary theoretical explorations reported that the direction-dependent CEP permeability might be partly controlled by the sharp composition gradients found in the CEP (Noailly and Lacroix, 2012). Overall, it seems highly relevant to consider the effects of CEP composition changes on the poromechanical properties of the tissue and on the simulation of mechano-transport phenomena and cell nutrition in the IVD. While the present report tackles this issue, we additionally hypothesize that extra ECM depletion in the CEP can trigger early DD process through indirect mechano-transduction phenomena.

## Materials and Methods

### Modeling Spatial CEP Heterogeneity

**FIGURE 1:**
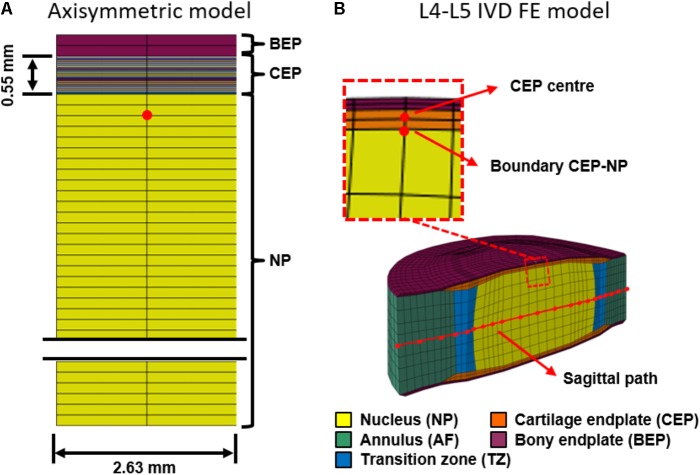
2D and 3D Finite element models. **(A)** 2D axisymmetric cartilage endplate (CEP) model with surrounding tissues: bony endplate (BEP), and nucleus (NP). The CEP includes different composition layers. The red dot highlights the point of analysis in the 2D model. **(B)** 3D L4-L5 composition-based IVD model including NP-AF transition zone (TZ). The red dot-line is the sagittal path used to evaluate the solute concentrations. The zoom area highlights the zone through which the mass loss was analyzed (See **Figure 6**), and the red dots are the points of analysis: CEP center and NP-CEP boundary.

An axisymmetric finite element (FE) model was created to represent a central plug of the CEP underlaid with NP tissue up to the IVD center so as to simulate a reserve of water within the disk. A top layer of BEP was also included (**Figure 1A**). The CEP and the NP were both modeled as osmo-poro-hyperelastic materials with explicit consideration of the proteoglycan fixed charge density (c_F_), total water content (n_F_), and collagen content (ρ_c,tot_) (Schroeder et al., 2007). The BEP was considered as a linear poroelastic material (Malandrino et al., 2011). NP composition corresponded to that of a healthy disk, and was taken from Ruiz Wills et al. (2016). As for the CEP, the model considered either a gradient of composition from the NP to the BEP (Roberts et al., 1989) or a virtually homogenized mean composition.

### CEP Composition Permeability Approach

Motivated by experimental evidences (Comper and Lyons, 1993) and by a previous pilot numerical study (Noailly and Lacroix, 2012), a new composition-dependent permeability formulation was established to fully capture the effect of tissue composition on the effective fluid permeation through the CEP. A pre-existing strain-dependent permeability model (Schroeder et al., 2007) was used and modified to incorporate the effect of collagen and proteoglycan (Equation 1):

(1) $$\kappa_{\mathrm{CEP}}=A\cdot exp(B.c_{F}+C\cdot \rho_{\mathrm{c,tot}})\cdot {\left(\frac{1-n_{F0}}{1-n_{F}}\right)}^{M}$$

where κ_CEP_ is the tissue hydraulic permeability in mm^4^/Ns, and *A* is a zero-strain permeability value, for a reference composition defined by parameters *B* and *C*. *B* and *C* respectively, rule the proteoglycan- and collagen-dependence n_F0_ is the initial water content at zero strain, and *M* is a model parameter that controls the rate of permeability change along tissue consolidation.

To determine *A, B*, and *C*, a similar dependence of κ_CEP_ upon composition for both the CEP and the NP was assumed and two sets of matching composition/permeability values available for the NP were used (**Table 1**). The method of least squares was used to find the set of parameters that minimize the difference between a mean κ_CEP_ value calculated through Equation 1 and CEP permeability values reported in modeling (Malandrino et al., 2009). The mean κ_CEP_ was calculated by using mean CEP composition parameters taken from the experimental measurements of Roberts et al. (1989). The collagen content was obtained from hydroxyproline data using the expression reported by Neuman and Logan (1950):

(2) %Collagen=%Hydroxyproline⋅7.46

The glycosaminoglycans content was determined through the reported quantity of dimethylmethylene blue according to the method reported by Farndale et al. (1986), and the fixed change density was further calculated using the formulation reported by Narmoneva et al. (1999).

**Table 1:** Data used to find the parameters A, B, and C from Equation 1 though constrained optimization.

Property	Constraints		Criteria for constrained optimization
		
	Nucleus^∗^		Cartilage endplate^∗∗^
		
	Healthy	Degenerated	Mean composition
Fixed charge density (mEq/mL)	0.3	0.23	0.17
Water content (fraction of wet weight)	0.8	0.76	0.66
Collagen content (fraction of dry weight)	0.09	0.285	0.24
Permeability (mm^4^/Ns)	0.0009	0.0045	0.0025–0.015


*^∗^Nucleus healthy and degenerated properties taken from Ruiz Wills et al. (2016). ^∗∗^Cartilage endplates fixed charge density from Roberts et al. (1989), water and collagen content from Fields et al. (2015) and permeability values from Malandrino et al. (2009).*

### 3D Composition-Transport-Cell Viability Disk Model

A L4-L5 3D FE disk model was taken from a previous studies, e.g., tissue distribution and mesh density from Ruiz et al. (2013), and constitutive equations and composition parameters from Ruiz Wills et al. (2016). The IVD model included all disk tissues, i.e., AF, NP, transition zone (TZ), CEP and BEP (**Figure 1B**). The BEP poroelastic formulation used in the 3D model was the same to that used for the axisymmetric model, and the poromechanical material properties were taken from the literature (Malandrino et al., 2011).

The CEP permeability was considered composition-dependent using Equation 1. AF and NP permeability were strain-dependent according to the following expression (Schroeder et al., 2007):

(3) $$\kappa =\alpha {\left(1-n_{\mathrm{exf}}\right)}^{-M}$$

where α stands for an initial permeability at zero strain, n_exf_ is the extra-fibrillar water content, and *M* is a positive constant that controls volumetric strain dependency, as in Equation 1. The viscoelastic fibers of the AF were also included. As for the solid matrix of the CEP, a composition-based formulation similar to the one used for the AF and the NP (Ruiz Wills et al., 2016) was adopted. The disk model was fully coupled to the metabolic-transport model (Ruiz Wills et al., 2016).

### Cell Viability Criteria

The cell viability model was validated in a previous study (Malandrino et al., 2015b). The model considered the effect of pH and glucose on cell density by assuming that cells start to die when: a) glucose level was below 0.5 nmol/mL, and/or b) pH decreased below 6.8 (Horner and Urban, 2001; Razaq et al., 2003; Bibby and Urban, 2004; Guehring et al., 2009). Under the aforementioned unfavorable nutritional environment and pH, the cell density decreased exponentially with time according to the following expression:

(4) $$\rho_{\mathrm{cell}}=\rho_{\mathrm{cell},0}\cdot \exp[-(\alpha_{\mathrm{glucose}}+\alpha_{\mathrm{pH}})t]$$

where ρ_cell_ and ρ_cell,0_ are the current and initial cell density, respectively, the death rate (α_pH_) due to low pH was constant and equal to 3.43 × 10^-6^s^-1^ according to the cell experiment of Horner and Urban (2001). The death rate (α_gluc_) due to low glucose concentrations was calculated according to the formulation proposed by Zhu et al. (2012) in function of the current glucose concentration (C_gluc_):

(5) $$\alpha_{\mathrm{gluc}}=\left(\frac{C_{\mathrm{gluc}}-0.5}{C_{\mathrm{gluc}}+0.2}-\frac{\left|C_{\mathrm{gluc}}-0.5\right|}{C_{\mathrm{gluc}}+0.2}\right)$$

### Boundary Conditions and Calculations

In the 2D model, sequences of rest and day activity were approximated through 1h and 2h of 0.26 and 0.78 MPa compressive loads, respectively. The load was applied on the BEP. The bottom of the NP was fixed and impermeable. The model was laterally confined, in terms of both lateral displacements and fluid velocities. The transported fluid mass (m_s_) per unit of area, in Kg/m^2^, was calculated as the integration of the fluid velocity (v_f_) along the time as shown in Equation 5, where ρ_f_ is water density in Kg/m^3^. Both m_s_ and v_f_ were evaluated at different points: NP area and CEP area (**Figure 1A**). Positive fluid velocity values indicated that water travels from the NP to the BEP.

(6) $$m_{s}=\rho_{f}\int v_{f}dt$$

In the 3D model, 3 days of daily load, i.e., 8 h of rest under 0.11 MPa of compression followed by 16 h of activity under an average pressure of 0.54 MPa (Wilke et al., 1999), were simulated. The lower BEP was fixed in all directions, and the pressure was applied at the upper BEP. The external pressure was considered atmospheric. Solute concentrations were applied at the edges of the AF and CEP. All tissue composition and solute concentrations were taken from the literature (**Table 2**) (Roberts et al., 1989; Dao et al., 2014; Ruiz Wills et al., 2016).

**Table 2:** Material properties used for the different disk tissues in the 3D model.

Parameter	Tissue					
	
	Nucleus^∗^		Annulus^∗^		Cartilage endplates^∗∗^	
			
	Healthy	Degenerated	Healthy	Degenerated	Healthy	Degenerated
Matrix shear modulus (MPa)	1	0.8	1	0.7	1	0.8
Initial fixed charge density (mEq/mL)	0.30	0.23	0.20	0.20	0.17	0.13
Initial water content (% of wet weight)	80	76	75	70	66	60
Collagen content (% of dry weight)	15	28.5	65	78	24	35
External salt concentration (mEq/mL)	0.15	0.15	0.15	0.15	0.15	0.15
α (mm^4^/Ns)	0.00016	0.00045	0.00016	0.00045	0.0017	0.044
Constant M (-)	1.2	0.9	1.2	0.9	8.5	8.5


^∗^ Nucleus and annulus healthy and degenerated properties taken from Ruiz Wills et al. (2016). ^∗∗^ Cartilage endplates shear modulus taken from nucleus values in Ruiz Wills et al. (2016), initial fixed charge density from Roberts et al. (1989), α from Equation 1, and composition parameters from Fields et al. (2015).

The effects of different endplate conditions were explored: a) grade I (healthy) CEP, b) grade III (degenerated) CEP, grade I AF and NP, and c) grade III NP, AF, and CEP (Pfirrmann et al., 2001). For each simulated condition, cell viability, mass flow and solute concentrations were evaluated at the mid sagittal plane (**Figure 1B**).

## Results

### Composition-Dependent CEP Parameters

**FIGURE 2:**
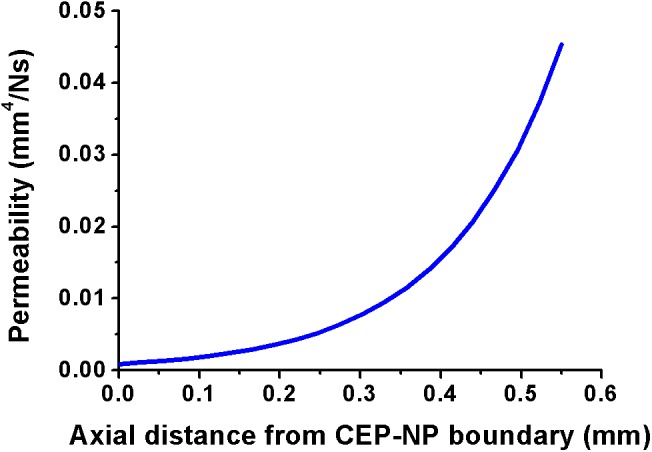
Cartilage endplate (CEP) permeability gradients obtained within the 2D FE model through Equation 1. The horizontal axis corresponds to the thickness of the CEP, from the NP to the BEP. From Ruiz Wills (2015) with kind permission from the author.

The back-calculated parameters for the composition-dependent permeability formulation were: A = 0.891mm^4^/Ns, B = -22.99 mL/mEq, and C = -0.00012. The obtained parameters *B* and *C* had negative values, and the absolute value of the former was much larger than that of the latter. Using these parameters in Equation 1 and simulating a gradient of composition (water, proteoglycan and collagen contents) from the NP to the CEP led to the CEP permeability distribution shown in **Figure 2**, where the permeability increased from the NP to the BEP. The maximum permeability value was 0.045 mm^4^/Ns and the minimum value was 0.0011 mm^4^/Ns. The maximum values of permeability was one order of magnitude higher than the minimum according to previous review of CEP permeability values reported in the literature Malandrino et al. (2009), where the maximum and minimum values of permeability were 0.0014 mm^4^/Ns and 0.0001 mm^4^/Ns, respectively.

**FIGURE 3:**
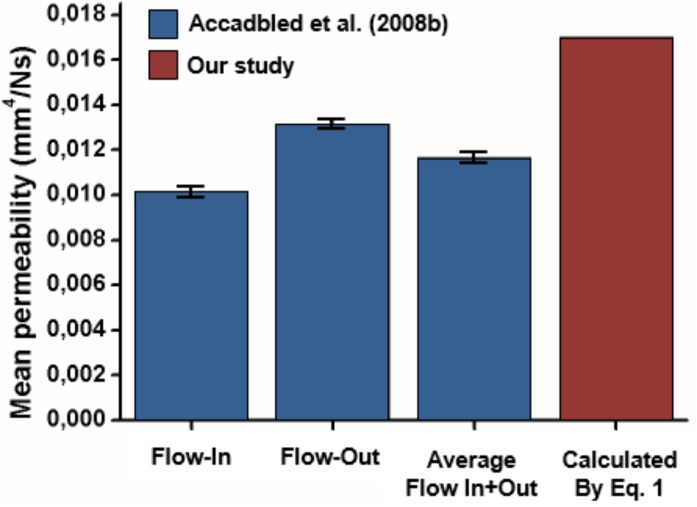
Comparison of the permeability value obtained by Equation 1 and experimental values: flow-in, flow-out, and average of flow-in and flow-out reported by Accadbled et al. (2008).

The permeability value obtained by using a mean CEP composition, calculated from the experimental study of Roberts et al. (1989), in Equation 1 with the back-calculated parameters *A, B* and *C*, was 0.017 mm^4^/Ns. This result is close to the mean value of 0.012 mm^4^/Ns measured by Accadbled et al. (2008) in central endplate samples (**Figure 3**). Using the composition values for a degenerated CEP the permeability was 0.044 mm^4^/Ns, representing an increment of a 59%. Both values, i.e., healthy and degenerated, were used as initial (i.e., low strain) homogeneous CEP permeability for the 3D model simulations.

### Axisymmetric Study

**FIGURE 4:**
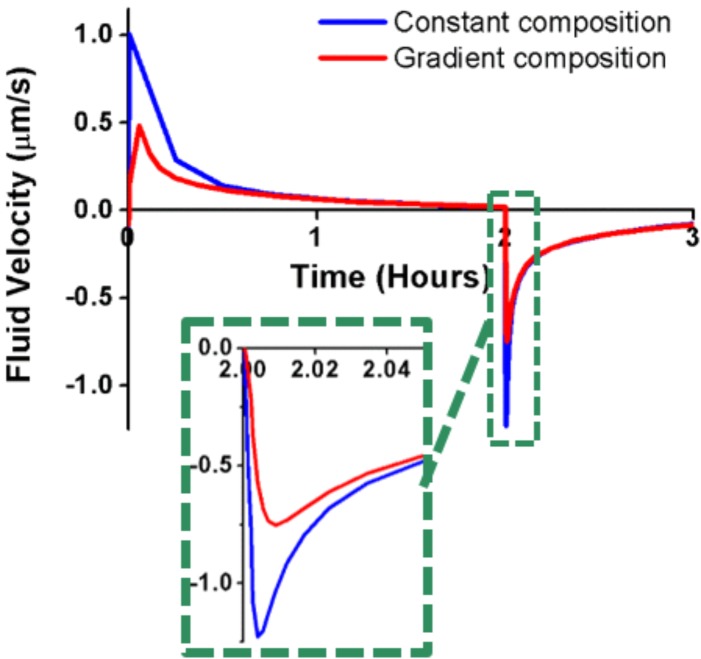
Fluid velocity in the NP, nearby the CEP (see Fig. 1), calculated through the 2D model by using a cartilage endplate (CEP) model with homogenized composition (blue line) and a CEP model with a gradient of composition (red line). The zoom area shows the attenuation of the fluid velocity by the composition gradient with sudden load changes.

**FIGURE 5:**
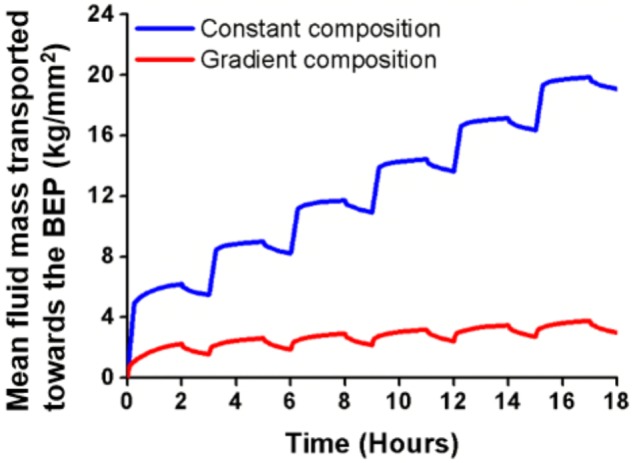
Mean fluid mass transported toward the BEP, calculated through the CEP of the 2D model for six cycles of compressive loads applied onto the BEP.

Compared to constant CEP permeability, the composition-based gradient of CEP permeability reduced by up to 55% the fluid velocity peaks in the vicinity of the NP-CEP boundary (2D model, red dot, **Figure 1**) when external loads suddenly changed from rest to active and from active to rest (**Figure 4**). Also, the decay of fluid velocity after the load application was slightly lower with the gradient of permeability (see zoom area in **Figure 4**). Additionally, the used of gradient composition in Equation 1, led to an average transport of fluid mass through the entire CEP, in direction to the BEP, much lower than the one obtained using constant composition, under cyclic loads (**Figure 5**).

### 3D Simulations

**FIGURE 6:**
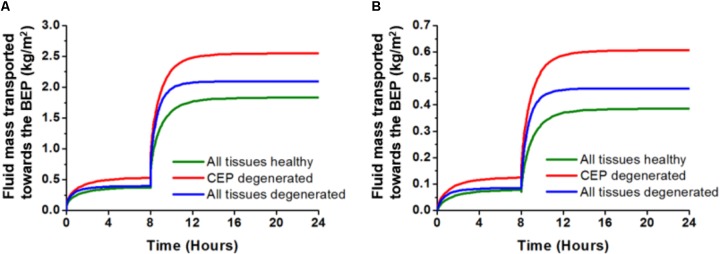
Results obtained with the 3D model for the three different tissue conditions. Fluid mass transported toward the BEP, calculated after three simulated days at **(A)** the CEP centre, and **(B)** the boundary CEP-NP with (i) all tissues simulated as healthy, (ii) only the CEP simulated as degenerated, (iii) all tissues, i.e., CEP, NP, and AF, simulated as degenerated. From Ruiz Wills (2015) with kind permission from the author.

The transported fluid mass toward the BEP, through the center of the CEP at the end of one simulated day for the 3D model, was 2.55 kg/m^2^ when the CEP properties were altered (**Figure 6A**). This value was 39% higher than the values obtained for healthy disk properties. For the disk with all tissue properties degenerated, the transported mass was 2.09 kg/m^2^ which represented an increase of 14% compared to the healthy disk. At the CEP-NP boundary, the outcomes were similar. In fact, the fluid mass transported toward the BEP increased by 56% when the CEP was simulated as degenerated and by 18% when all IVD tissues were simulated as degenerated (**Figure 6B**).

**FIGURE 7:**
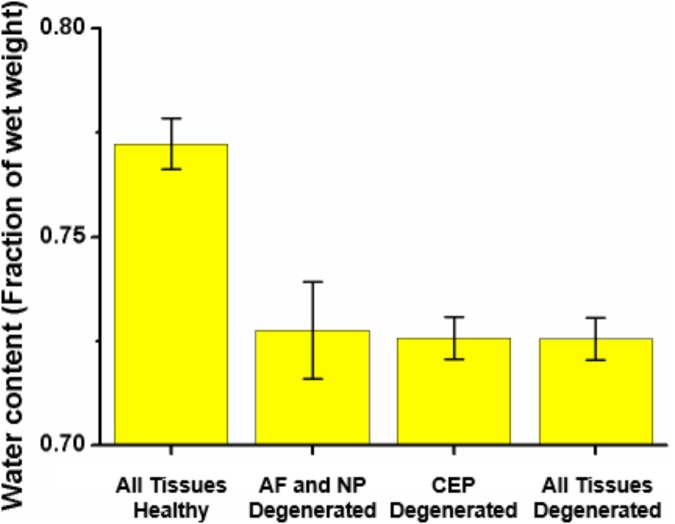
Mean water content obtained within the nucleus (NP) volume at the end of 16h of simulated activity under 0.54 MPa. The water content was calculated with (i) all tissues simulated as healthy, (ii) only NP and AF simulated as degenerated, (iii) only the CEP simulated as degenerated, (iv) all tissues, i.e., CEP, NP, and AF, simulated as degenerated.

The mean fraction of water content (porosity) at the end of one simulated day under 0.54 MPa computed within the NP volume was 0.772 ± 0.006 when the CEP was modeled as healthy. For the same simulated time and load condition, the degenerated CEP model made the mean water content in the NP drop to 0.726 ± 0.005, independently of whether the rest of the disk tissues were simulated as degenerated or not (**Figure 7**). A two-way ANOVA over the full set of NP nodes revealed that NP water contents were statistically different when the CEP was modeled as healthy or degenerated.

**FIGURE 8:**
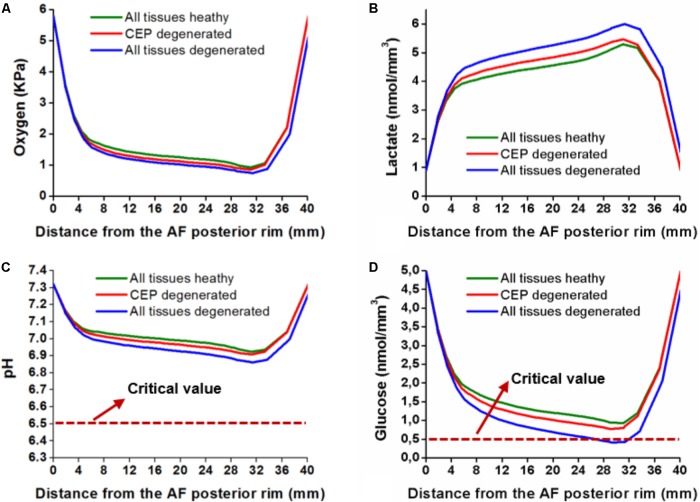
Solute concentrations distribution along the sagittal path of the 3D model after 3 days simulated. **(A)** Oxygen content, **(B)** Lactate production, **(C)** pH level; the dot line is the critical value for cell viability, and (d) Glucose concentration; the dot line is the critical value for cell viability. Curves are reported for (i) all tissues simulated as healthy, (ii) only the CEP simulated as degenerated, (iii) all tissues, i.e., CEP, NP, and AF, simulated as degenerated. From Ruiz Wills (2015) with kind permission from the author.

The 3D transport simulations revealed that all solute concentrations decreased when the CEP composition was that of a tissue considered as degenerated (**Figure 8**). In fact, the glucose concentration was reduced by 16% and the pH was decreased by 2.4%. The minimum values obtained were 0.77 nmol/mm^3^ and 6.90 for glucose concentration and pH, respectively, at the anterior TZ (**Figures 8C,D**). For a disk model with all tissues simulated as degenerated, the reduction of solute concentration was higher: the glucose concentration decreased by 55% and the pH decreased by 9%, with minimum values of 0.41 nmol/mm^3^ and 6.86 respectively (**Figures 8C,D**).

**FIGURE 9:**
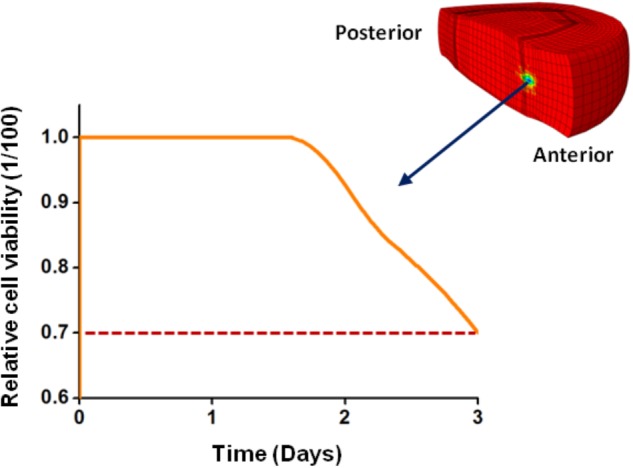
Cell viability in the anterior transition zone (TZ) along the 3 days simulated for a disk with all tissue degenerated. Cells started to die before the second day simulated finished. From Ruiz Wills (2015) with kind permission from the author.

All disk cells remained alive after the 3 days simulated when only the CEP was degenerated. However, the simulation of a disk with all tissues degenerated activated cell death. Cells started to die between the simulated days 1 and 2, and simulated CEP degeneration was a condition sine qua non to achieve nutrition-induced cell death. At the end of the third day, 70% of the cells remained alive (**Figure 9**).

## Discussion

### Evaluation of the Composition Permeability Formulation

In the vertebral endplate, the CEP has the strongest hydraulic resistance and most likely controls the effective permeability of the whole osteochondral construct (Rodriguez et al., 2011; Malandrino et al., 2014a). Hence the similarity of our CEP permeability value, calculated with mean composition parameter values, and the vertebral endplate permeability value measured by Accadbled et al. (2008) supports the validity of our new composition-dependent CEP permeability model and the calibration thereof. In addition, the negative signs obtained for parameters *B* and *C* reflect the correlations found by Williamson et al. (2001) for bovine articular cartilage, where increased proteoglycan and collagen contents led to reduced hydraulic permeability. Also, the relatively large B parameter value suggested that proteoglycans mostly control the effective CEP permeability, which illustrates nicely the control of bounded water by proteoglycan aggregates and the likely impact thereof on the effective transport of water molecules. Accordingly, Comper and Lyons (1993) showed that the frictional resistance to water flow in the articular cartilage is controlled by the proteoglycans. In contrast, Mahmoodian et al. (2011) found no significant correlation between cartilage permeability and proteoglycan content, yet only measured two samples.

According to our optimized *B* and *C* parameter values, the use of altered composition for the CEP led to an increased permeability value. This result contrasts with the study of Riches and McNally (2005) where the CEP permeability was found to decrease with DD. Yet, our results are supported by the measurements from Rodriguez et al., 2011 who found that the total endplate permeability increases with age (and with degeneration), and reported mobility measurements strongly suggest that total endplate permeability is rather controlled by the CEP. Interestingly, these findings contrasted with the paradigm that CEP permeability decreases with degeneration because of possible calcification. Actually, the effect of the calcification nodules, identified by Benneker et al. (2005a), on CEP permeability and disk nutrition remain poorly documented. Yet, these nodules should be visible on micro-CT, and the study of Malandrino et al. (2014a) suggested that their impact on nutrient transport, from the peripheral disk vasculature to the disk tissue, is limited.

### Axisymmetric Study

Permeability gradient through the CEP was largely responsible for the control of fluid flow by tissue composition. Interestingly, the composition-based permeability gradients reduced by half the maximum fluid velocities in the CEP under sudden changes of the mechanical loads. This outcome might be interpreted as a functional protection of chondrocytes against catabolic shift of cell activity because of chronic flow-induced shear strains during dynamic loading (Blain, 2007). Furthermore, compared to the results was obtained with a constant permeability based on a mean composition, results obtained with the permeability gradient reveal that the composition gradient could strongly limit the loss of fluid mass through the NP-CEP boundary along compressive load cycles. This effect was due to limited local consolidation of the tissues at the NP-CEP interface, directly promoted by the simulated gradient of permeability. This outcome is conceptually similar to the valve theory proposed by Ayotte et al. (2000, 2001), but where the local instantaneous stiffness of the tissue is controlled by the ability of the fluid to flow through elementary material volumes.

### 3D Simulations

In the 3D model, the calculated exchange of fluid mass through the CEP was dramatically affected by the simulated degeneration of the tissue (**Figure 5**). In fact, the largest fluid mass loss calculated in the NP was already achieved when only the CEP was simulated as degenerated. Interestingly, (Rodriguez et al., 2011) reported a non-linear decrease of the proteoglycan contents with age nearby the endplates, with a strong decrease from 30 to 45 years of age. Accordingly, our model suggests that decreased CEP permeability, and chronic dehydration of a healthy NP under mechanical loads, is part of an early degeneration process. In contrast, Benneker et al. (2005a) reported that significant endplate occlusion would rather occur in advanced degeneration, if we define the level significance as about 50% of occlusion, i.e., sufficient occlusion to generate severe nutrient deprivation in the disk, according to previous numerical simulations (Zhu et al., 2012). Actually, among the tissues affected by degeneration in the intervertebral space, the CEP is the first structure to show alterations in radiological signals (Benneker et al., 2005b). In Rodriguez et al. (2011), the loss of GAG in the disk correlated with an increase of CEP porosity. Curiously, it did not correlate with an increase of CEP mobility, probably because of tissue consolidation problems during the permeation experiments. Hence, our results let us suppose that early CEP degeneration allows the fluid to move faster through the vertebral endplate, producing a large exchange of water that leads to an increased poromechanical consolidation of the rest of the disk tissues.

Bone micromechanical properties can be estimated through ultrasounds (Hellmich and Ulm, 2005) and micromechanical models suggest that the microscale lacunar porosity of bone influences the transport of fluid through the tissue (Scheiner et al., 2016). Especially, Scheiner et al. (2016) pointed out that the pore pressure that develops at the lacunar level, under mechanical loads, can be involved in bone mechanobiology. Malandrino et al. (2014a) found that BEP permeability and porosity can vary greatly from one BEP to another or even among different locations within one BEP, due to structural variations at the extravascular level. Such a variability might affect the response of the basic multicellular units, at the micrometer scale, responsible for macroscopic bone remodeling, as reflected by the BEP density and mechanical measurements reported by Grant et al. (2002). Malandrino et al. (2014a) reported that variations in BEP structure and macroscopic poromechanical properties had no effect either on CEP consolidation, or on the transport of nutrients and metabolites through the vertebral endplate. Yet, the authors calculated that fluid velocities in the CEP might locally change depending on the BEP properties, which could, in turn, affect the CEP chondrocyte biology (Blain, 2007) and have an influence on the biological interplays that exist between tissues in osteochondral systems (Suri and Walsh, 2012). Hence, the effect of BEP microstructure and biology on CEP tissue fate should be further explored. As for whether the CEP can affect the mechanobiology and structure/composition of the BEP, we found minor relative differences, i.e., 1.3%, in terms of pore pressure calculated in the BEP center, with healthy and CEP degenerated. The calculation of fluid velocities gave similar outcomes, suggesting that poromechanical CEP alterations might have no relevant effect on the BEP.

Under mechanical loads, the amount of water inside the nucleus was decreased by 6% when the CEP was considered as degenerated. Accordingly, such reduction in NP water content affected the transport of solutes. Indeed previous simulations reported by Ruiz Wills et al. (2016) revealed that a reduction of 4% in NP water content, i.e., with a change from grade I to grade III, significantly reduced the glucose and oxygen concentrations in the IVD. In the present study, the altered CEP composition mostly reduced the glucose concentration at the anterior TZ (**Figure 7D**). The consolidation of the NP was not homogeneous, and the reduction of nutrient nearby the AF was associated with an increased consolidation and loss of tissue porosity nearby the NP-CEP interface. This localized tissue compaction generated a physical barrier to the diffusion of solutes from the CEP to the most remote disk areas of the IVD.

The levels of pH and glucose obtained with a CEP simulated as degenerated were lower than the levels found when the AF and NP were simulated degenerated, separately or together. The fact that the CEP had a stronger effect on the solutes highlights the likely importance of this tissue in the possible involvement of indirect mechanotransduction in early DD. Indeed, longitudinal studies in human volunteers support the hypothesis that damage to the CEP is the triggering factor for Modic change and DD (Määttä et al., 2016). Several mechano transport IVD models reported in the literature point out the CEP as a path for nutrition, and indicate that CEP calcification has a larger influence on solute concentration (Ferguson et al., 2004; Mokhbi Soukane et al., 2009; Malandrino et al., 2014a,b). But as analyzed in the introduction of the present paper, if CEP calcification has any impact in DD, it would be rather involved at advanced stages of degeneration. In contrast, our present results suggest that early CEP degeneration produces chronic NP dehydration under mechanical loads, which influences disk cell nutrition and might lead to locally severe cell nutritional stress, and even cell death as DD progresses. Assuming that nutritional stress at the TZ triggers a catabolic shift of ECM turnover (Neidlinger-Wilke et al., 2012), the present results might provide educated explanations about why AF damage is not necessarily a consequence of ECM bulk degradation in the NP (Lama et al., 2013).

Our new simulations-based hypothesis largely relies on the early depletion of the proteoglycans of the CEP and on the consequence thereof on the tissue permeability. Arguably, a recent study reported that water and glycosaminoglycan (GAG) contents do not change with CEP degeneration (DeLucca et al., 2016). The authors even measured an increase of fixed charge density in the CEP with degeneration. Yet, such increment was suggested to come from fragments of NP GAGs that become temporally entangled to the CEP, and an initial increase of CEP permeability with degeneration might also explain the migration of GAG from NP to CEP. Indeed, DeLucca et al. (2016) found that CEP permeability decreases once the NP start to degenerate. Our simulations would represent an earlier process, i.e., early CEP degeneration with a healthy NP. Indeed, endplate-driven DD has been reported to start before 30 years of age, which can be classified as a relatively early degeneration process (Adams and Dolan, 2012). Discussing the possible causes of early CEP degeneration is beyond the focus of the present study and requires further *in silico*- and evidence-based information.

As any numerical study, the present study has limitations. Firstly, it only considered two sequences of sustained compression loads (night rest and day activity) to simulate daily loading, in the 3D simulations. Within a regular day the disk is subjected to different static and dynamic load variations at different frequencies, the orders of magnitude of which range from 0.10^-1^ to 0.10^1^ Hz. However, Malandrino et al. (2011) showed that sustained compression affects disk nutrition the most compared to cyclic loads, as far as indirect mechanotransduction is concerned. Secondly, tissue average contents for all disk biochemical components were considered. Thirdly, the geometry of the 3D disk model remained generic. Patient-specific geometries might give different shapes of disks and tissue regions and could provide further information about the influence of the CEP on disk degeneration combined with the effects of different sets of diffusion distances (Malandrino et al., 2015b). Fourthly, the initial cell density was not changed when all disk tissues were considered degenerated. Such consideration is out of the scope of this study since our simulations evaluates the nutrition environment in the disk before the cells started to die. Finally, because of computational cost, a mean homogenized CEP permeability was considered for all disk tissue in the 3D simulations, impeding assessing the impact of the CEP composition gradient on disk cell nutrition. Yet, our homogenized value, calculated from mean composition measurements through our permeability model, could be contrasted with the experimental measurements reported by Accadbled et al. (2008).

**FIGURE 10:**
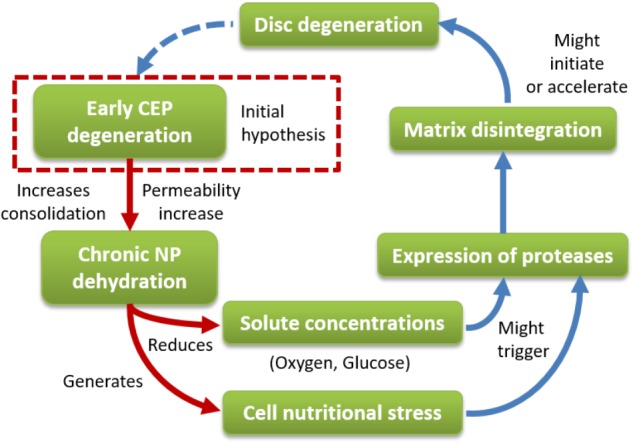
Disk degeneration workflows including the contribution of cartilage endplate (CEP) degeneration. Our initial hypothesis is that early CEP degeneration generates chronic dehydration of the NP under mechanical loads. The fall of water in the NP reduces the solute concentration and generates cell nutritional stress nearby the TZ. Such reduction might trigger the expression of proteases that might disintegrate the disk matrix and eventually initiate/accelerate disk degeneration. The red arrows represent the findings of the current study.

The results of the present study have led us to identify an alternative and rational way to explain the fall in disk nutrition in early DD, and to formulate a hypothesis about involvement of the CEP. The chronic dehydration of the healthy NP under mechanical loads when the composition of the CEP is altered is based on strong physical rationales. The consequent decrease of the level of glucose and pH at the NP-AF boundary might lead to downregulated expressions of matrix proteins and/or catabolic cell activities, e.g., through inflammation and matrix metalloproteinase (MMP) activity, (**Figure 10**). For example, MMP-7 and MMP-13 were reported, among others, to increase in early DD (Vo et al., 2013). Interestingly, MMPs able to cleave proteoglycans and collagen type II might produce a biochemical weakening of the inner AF structure. Such weakening might contribute to generate the microfractures observed in cadaveric specimens (Adams, 2004) and more recently reported in both clinical patients (Rajasekaran et al., 2008) and twin volunteers (Määttä et al., 2016). Extending the 2D model permeability gradient to the 3D IVD model may provide a better idea of the influence of the CEP structure and regulation of interstitial fluid velocities on the mechanoregulation of CEP chondrocytes and local release of inflammatory factors that, combined with Modic changes, might shed light on specific onsets of low back pain and DD (Zhang et al., 2008).

Regarding the clinical application of our findings, these strengthen the idea that CEP, instead of NP or AF, is the optimal target tissue for therapies in individuals where early DD and progression of DD leads to increasing clinical problems (Pereira et al., 2016). On one hand, stem cell or genetic interventions directed to NP/AF have not been successful (Sivakamasundari and Lufkin, 2013; Richardson et al., 2016; Vadalà et al., 2016). On the other hand, these interventions require invasive procedures through the AF that may bring along more negative than positive effects, as suggested by the adverse effects of discography (Carragee et al., 2009). Instead, the CEP as target tissue is accessible through transpedicular approach, e.g., for early regeneration through stem cells therapy (Pereira et al., 2016). Importantly, our results are in agreement with recent observations highlighting the role of endplate defects in Modic changes (MC) and DD (Eskola et al., 2012). However, we do not know which kind (morphology, biochemical composition, localization within CEP) of defects are harmful. In particular, we need basic research (including simulation models and AI) to explore the development of CEP alterations on the pathway to symptoms and altered structural changes.

The present study illustrates the concept of mechanistic simulation systems to improve the understanding of disk disease mechanisms. Such systems would support improved clinicians and patient information to better target and optimize therapies and avoid over-medicalization (Buchbinder et al., 2018). Furthermore, disease mechanism models might be efficient tools in transferring knowledge to other diseases. For example, modic changes/endplate-driven disk degeneration are the hallmark of severe (Määttä et al., 2015) and relatively early chronic LBP (Adams and Dolan, 2012) and share many similarities with osteoarthritis (OA) (Dudli et al., 2016). They are associated with genetic variants of Pro-inflammatory cytokine Interleukin (IL)-1 (Karppinen et al., 2009) that have also been suggested to favor the extension of OA problems in a patient through sensitization of the immune system (Meulenbelt et al., 2010).

## Conclusion

In summary, this study presents a novel approach to simulate the composition-dependency of the permeability of the CEP to evaluate the regulatory function of this tissue in terms of fluid transport between the IVD and the adjacent vertebrae and the possible consequences on disk cell nutrition. The results of the 2D model showed that the very particular distribution of macromolecules is likely to influence largely the effective mobility of water through the CEP, adding to the capacity of the tissue to limit interstitial fluid velocity and retain water in the NP, under mechanical loads. Further alteration of the CEP composition in the 3D model, based on reported composition measurements, allowed evaluating the possible importance of the CEP in DD. Results suggest an alternative pathway to DD: early alterations of the CEP compositions makes the healthy NP lose increased amount of water under mechanical loads which hinders the transport of nutrients to the TZ. The fall in solute concentrations might activate the release of enzymes that disintegrate the disk ECM, possibly (i) increasing the risk of AF damage, (ii) initiating DD, or (iii) accelerating existing DD through nutrition-induced cell death. This statement identifies the CEP as a key factor in DD, which paves the way for guided experimental/clinical research about DD risk factors and the spatio-temporal mechanisms of DD in the young to middle-aged population.

## Author Contributions

JN designed the study. BF performed 2D simulations. CRW performed 3D simulations, reported the results, and wrote the manuscript. JK and MGB critically reviewed the manuscript and contributed to the discussion, CRW and JN discussed the results and commented on the manuscript at all stages.

## Conflict of Interest Statement

The authors declare that the research was conducted in the absence of any commercial or financial relationships that could be construed as a potential conflict of interest.
